# Retraction: Calcium Channel Blockers and Risk of Breast Cancer: A Meta-Analysis of 17 Observational Studies

**DOI:** 10.1371/journal.pone.0198220

**Published:** 2018-05-23

**Authors:** The PLOS ONE Editors

Following publication of this article [1], concerns were raised about errors in the article and the scientific soundness of the work. The *PLOS ONE* Editors had the article reassessed by a member of our Editorial Board and one of the journal’s Statistical Advisors with expertise in meta-analyses. This assessment confirmed that there are reporting errors in the published article as well as concerns about the study design, data analyses, and conclusions.

Specific concerns and errors include:

- The authors did not report the dates of inclusion for the literature search, and they did not specify whether language exclusions were applied in the literature search.
- Reasons for exclusion listed in the flow diagram are vague, presenting a concern about reproducibility.
- Holmes *et al*. (2013) is a population-based retrospective cohort study assessing association between medications and survival, but did not assess cancer development or risk. Thus, it should not have been included in the current meta-analysis.
- In Figure 2, two studies listed in Table 1 are not included in the cohort subgroup meta-analysis (Hole *et al*. 1998, Saltzman *et al*. 2013), and four studies listed in Table 1 are not included in the case-control subgroup analysis (Davis *et al*. 2007, Assimes *et al*. 2008), Li *et al*. 2003, Li *et al*. 2013). The reasons for excluding these articles are not explained by the authors. Li *et al*. (2013) is a case-control study but was included in the cohort subgroup in the analysis shown in Figure 2.
- Table 1 lists incorrect citation numbers for the last 7 articles listed.
- The number of cases was incorrectly reported for Fryzek *et al*. (2006) in the >5 years subgroup analysis in Figure 3 (438 instead of 4381), and the numbers reported for this study in substrata are total subject numbers instead of subgroup numbers.
- Numbers reported in Figures 2, 3 for Li *et al*. (2003, 2013) do not correspond to those reported in the primary literature. The data presented in Figures 4 and 6 for Li *et al*. (2013) were not found in the primary article.
- Lobular and ductal cancer cases from Li *et al*. (2013) were grouped in Figure 2.
- Different values for subtotal estimate (SE) and confidence intervals are reported for the “ever used” subgroup in Figure 3 versus in the Abstract and Results section.
- In the Results section, “Quantitative Summary (Meta-analysis)”, the authors report a “non-significant inverse association in case-control studies (RR = 0.98, 95% CI: 0.86, 1.09) [P = 0.02].” This statement is misleading, as the pooled estimate (RR = 0.98) indicates a neutral result. The meaning of the P value provided here is unclear.
- Data provided in the quantitative summary in Figure 6 does not match the corresponding data reported in the Results section.
- RR and 95% CI data presented in Figure 5 are not consistent with those shown in Figure 6.
- Only 12 of the 17 articles are included in the publication bias assessment, and the reason for excluding the remaining 5 is unclear.
- The Methods section statement beginning “When there was a significant publication with regard to CCB intake and breast cancer risk …” is misleading, as the trim and fill method was not reported as having been applied in this study.
- In Table 1, authors mention adjustments for sex in included studies, yet the meta-analysis reportedly included only female subjects.
- Concerns were raised regarding the use of a pooled risk ratio of only two studies, and regarding the pooling of risk ratios and odds ratios to derive an overall risk ratio.
- The authors pooled cohort and case-control studies in meta-analyses.
- The study lacked sensitivity analyses needed to adequately describe the results, including hospital-based vs population-based studies, studies with satisfactory adjustment sets vs. unsatisfactorily adjusted studies, high vs low quality studies, mortality vs incidence studies, leave-one-out-analysis, subgroup analyses by study region, or cancer stage.
- Concerns were raised about potentially misleading language in the description of Figure 4 results: Li *et al*. (2013) studies: " …for the 3 studies which followed patients for >10 years." This sentence gives the incorrect impression that the three studies presented in the figure are all cohort studies with a long follow up and implies that the controls were patients at the time of CCB assessment. This figure includes two cohort studies and a case-control study.
- Conclusions assert that this study found a “significant relationship with breast cancer”, but the estimates were predominantly indicative of neutral effects and did not achieve statistical significance.
- Conclusions also note that there was no indication of publication bias, but the authors did not discuss the limitations of tests used for analyzing small numbers of studies. Based on the results presented, the tests came close to statistical significance and some degree of bias was suggested by the funnel plot data.

In light of the above issues, the *PLOS ONE* Editors retract this article, as the integrity and validity of the study and its conclusions are compromised by the errors in reporting and analysis.

The authors could not be reached for comment.

## References

[1] Li W, Shi Q, Wang W, Liu J, Li Q, Hou F (2014) Calcium Channel Blockers and Risk of Breast Cancer: A Meta-Analysis of 17 Observational Studies. PLoS ONE 9(9): e105801 https://doi.org/10.1371/journal.pone.0105801 25184210 10.1371/journal.pone.0105801 PMC4153551

